# Supplementary material for: High-resolution positron emission microscopy of patient-derived tumor organoids
Source: Nat Commun. 2021 Oct 7;12:5883. doi: 10.1038/s41467-021-26081-6 (PMC8497512; doi:10.1038/s41467-021-26081-6)
Supplement: Supplementary file 1 — Supplementary Information [file 41467_2021_26081_MOESM1_ESM.pdf]

Supplementary Information

High-resolution positron emission microscopy of patient-derived tumor organoids

Syamantak Khan[1]†, June Ho Shin[2]†, Valentina Ferri[3], Ning Cheng[4], Julia E. Noel[2], Calvin Kuo[4], John B. Sunwoo[2], Guillem Pratx[1]*

1. Department of Radiation Oncology, Division of Medical Physics, Stanford University School of Medicine, Stanford, USA
2. Department of Otolaryngology, Division of Head and Neck Surgery, Stanford University School of Medicine, Stanford, CA, USA
3. Department of Radiology, Division of Nuclear Medicine and Molecular Imaging, Stanford University School of Medicine, Stanford, CA, USA
4. Division of Hematology, Department of Medicine, Stanford University School of Medicine, Stanford, CA, USA

† These authors contributed equally

* Corresponding Author email: pratx@stanford.edu

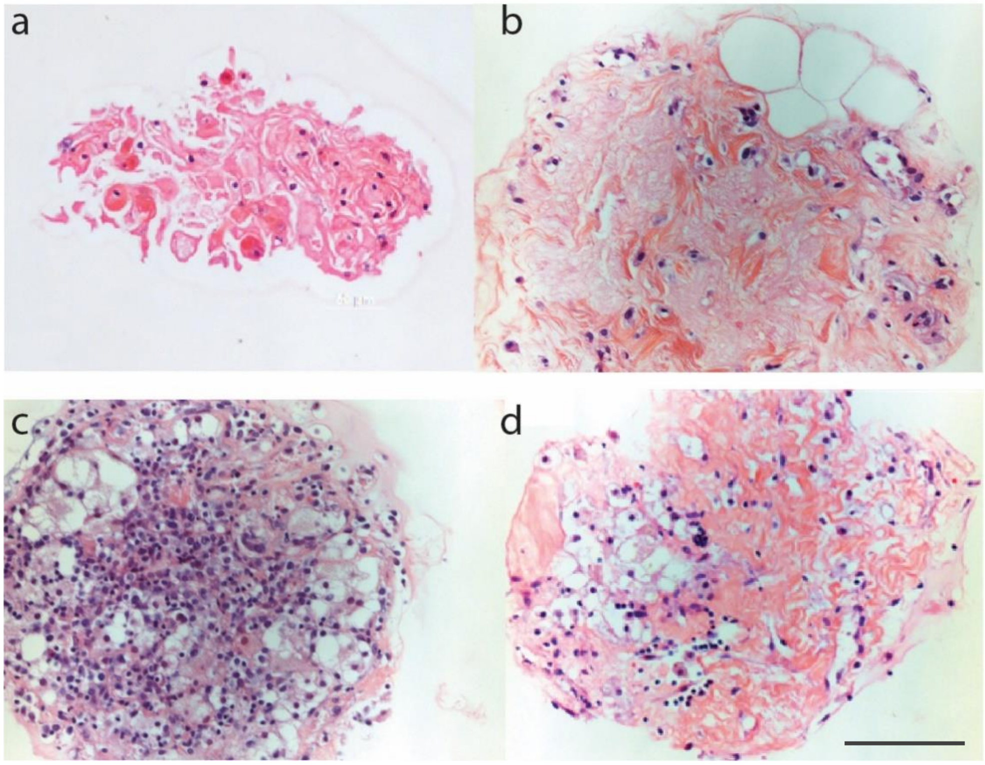

Supplementary Figure 1. Histological imaging of patient-derived tumor organoid of (a) squamous cell carcinoma from patient S1 and (b-d) papillary thyroid carcinoma from patient T1 shows heterogeneous tissue structure (20X magnification). Scale bar: 0.2 mm

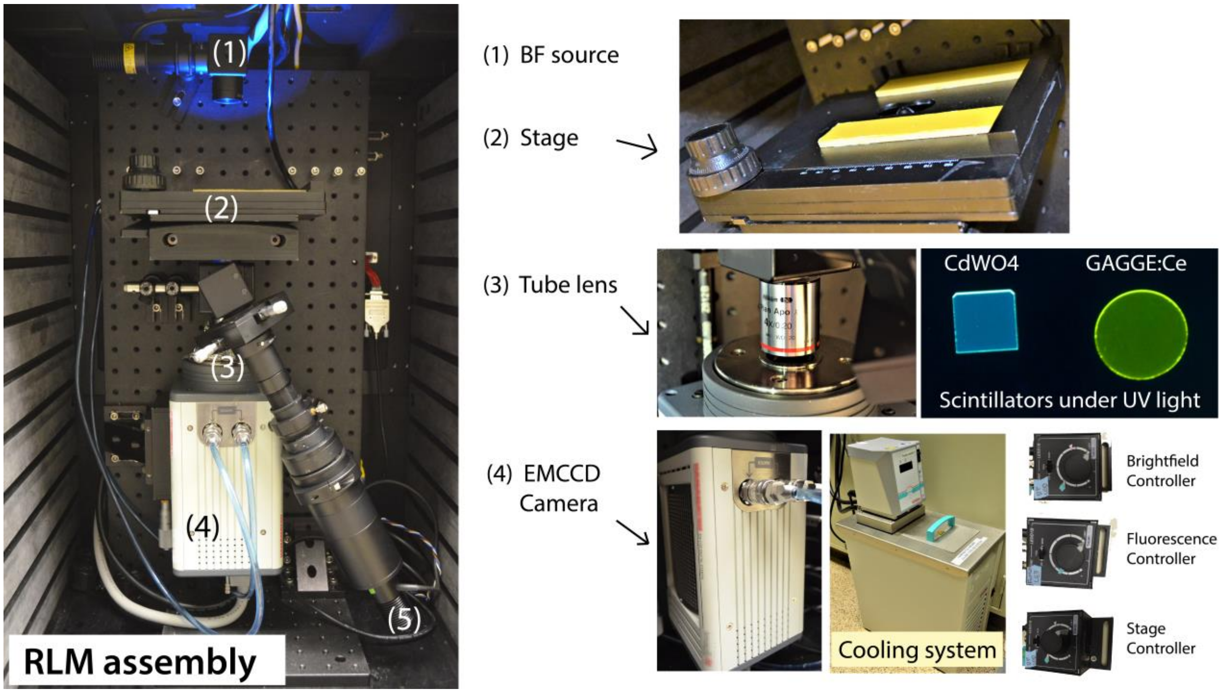

Supplementary Figure 2. Overview of the radioluminescence microscope. Left, a photograph of the fully assembled microscope, showing (1) brightfield illumination, (2) stage assembly, (3) tube lens connected to (4) EMCCD camera with a cooling unit, (5) LED light source for epifluorescence imaging. The optical train is composed of two microscope objective lenses aligned back to back. A 50-mm-focal-length lens (Nikon CFI Plan Apochromat λ 4X) is used in place of the standard f = 200-mm tube lens to demagnify the image and increase its brightness.

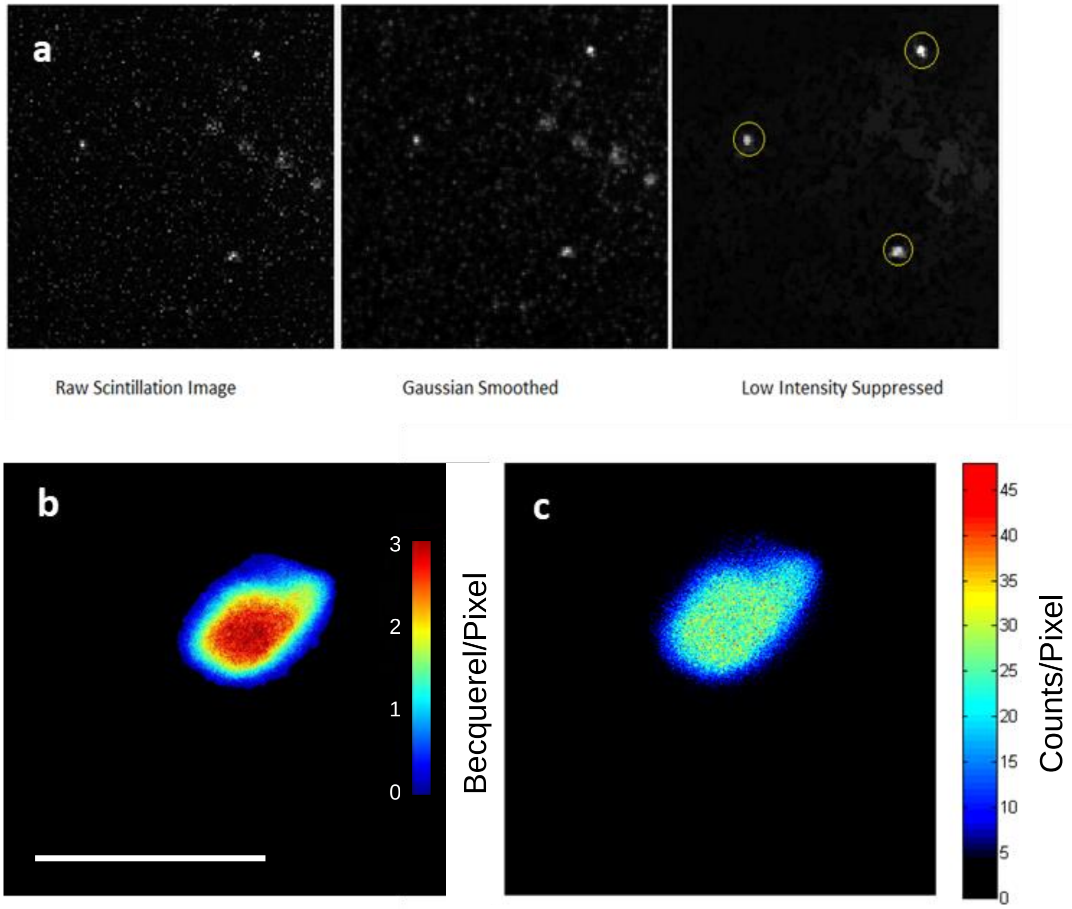

Supplementary Figure 3. Optical reconstruction of a single frame using ORBIT software package. (a) The serial image processing of the raw image (left) allows elimination of the background signal and reconstruction of three most reliable events from the frame. (b) Analog and (c) digital oPEM image of tumor organoid grown from patient T1.

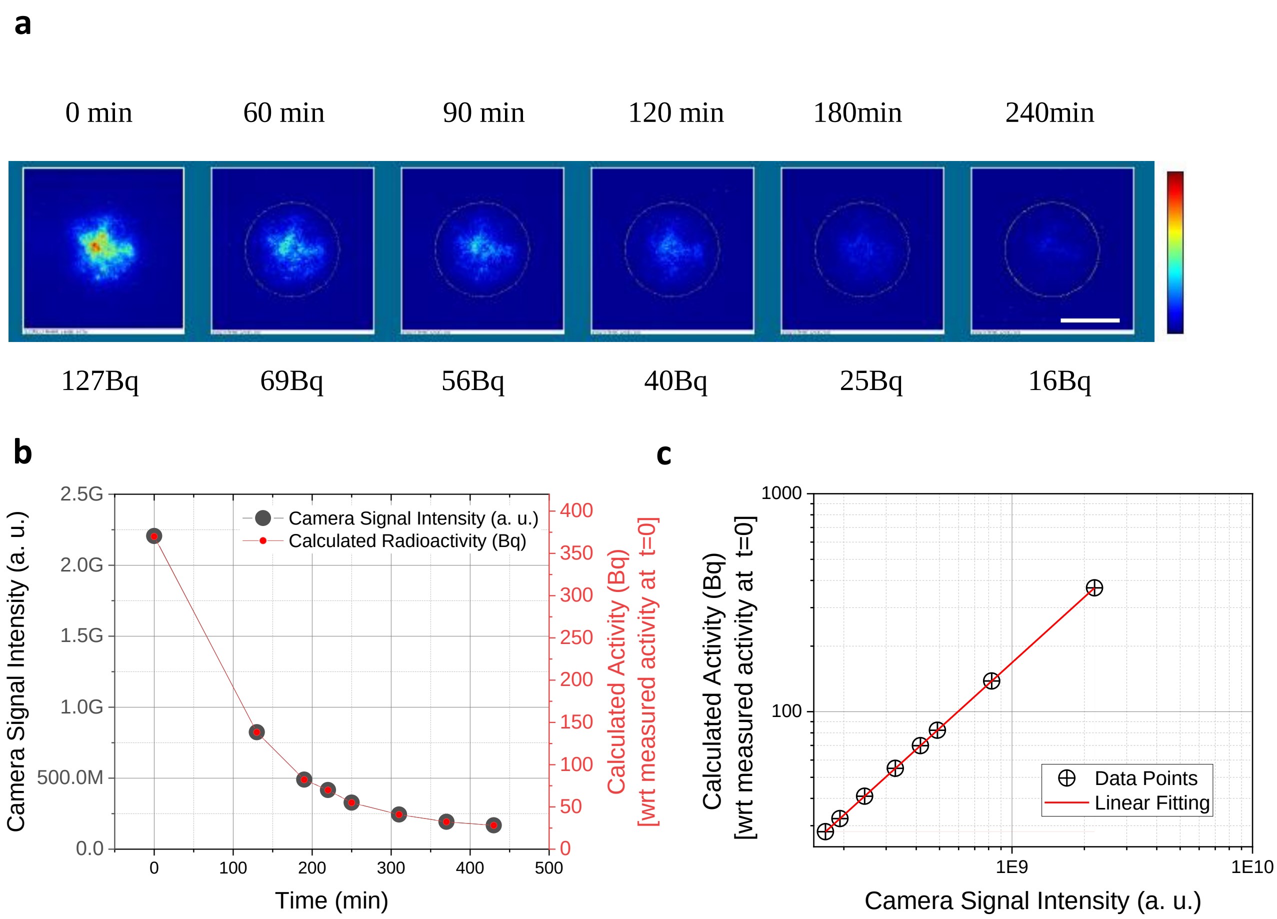

Supplementary Figure 4. Quantification of radioactivity from oPEM image. (**a)** oPEM image of a small FDG droplet with known activity. Image taken at different time points shows decreasing radioactivity. Color bar: Bq/pixel. Scale bar: 1mm (**b**) The time-dependent activity measured from the camera signal (within 1 mm^2) closely follows the decay-curve of ^{18}F (half-life of 110 min) (**c**) Calibration curve to convert camera signal into quantitative radioactivity measurements.

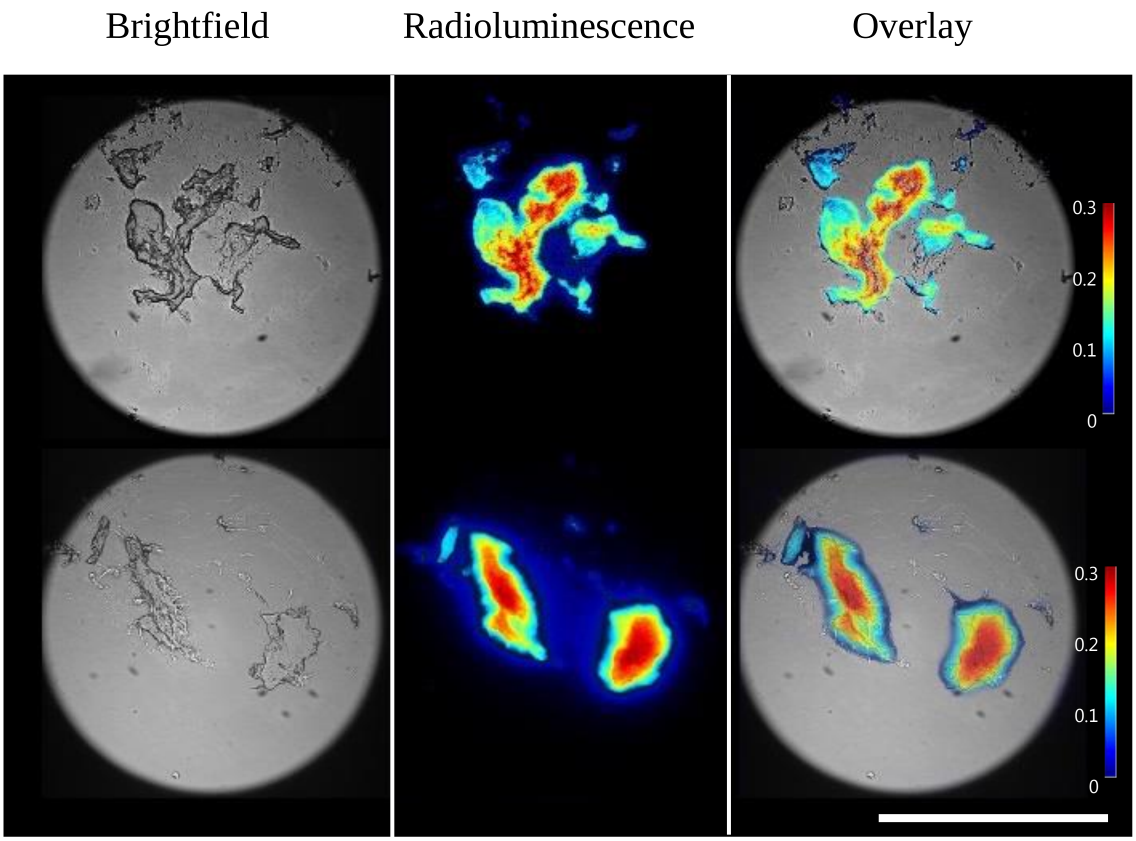

Supplementary Figure 5. oPEM of tumor organoids derived from patient S1. Brightfield (left) and radioluminescence (intensity colormap of FDG uptake, middle) images show hotspots indicating elevated metabolic activity in parts of the organoid structure in three different samples. The overlay (right) of brightfield and FDG images reveals spatial distribution of FDG uptake in further detail. Scale bar: 1mm. The intensity color bar (Bq/pixel) shows increasing FDG concentration from blue to red.

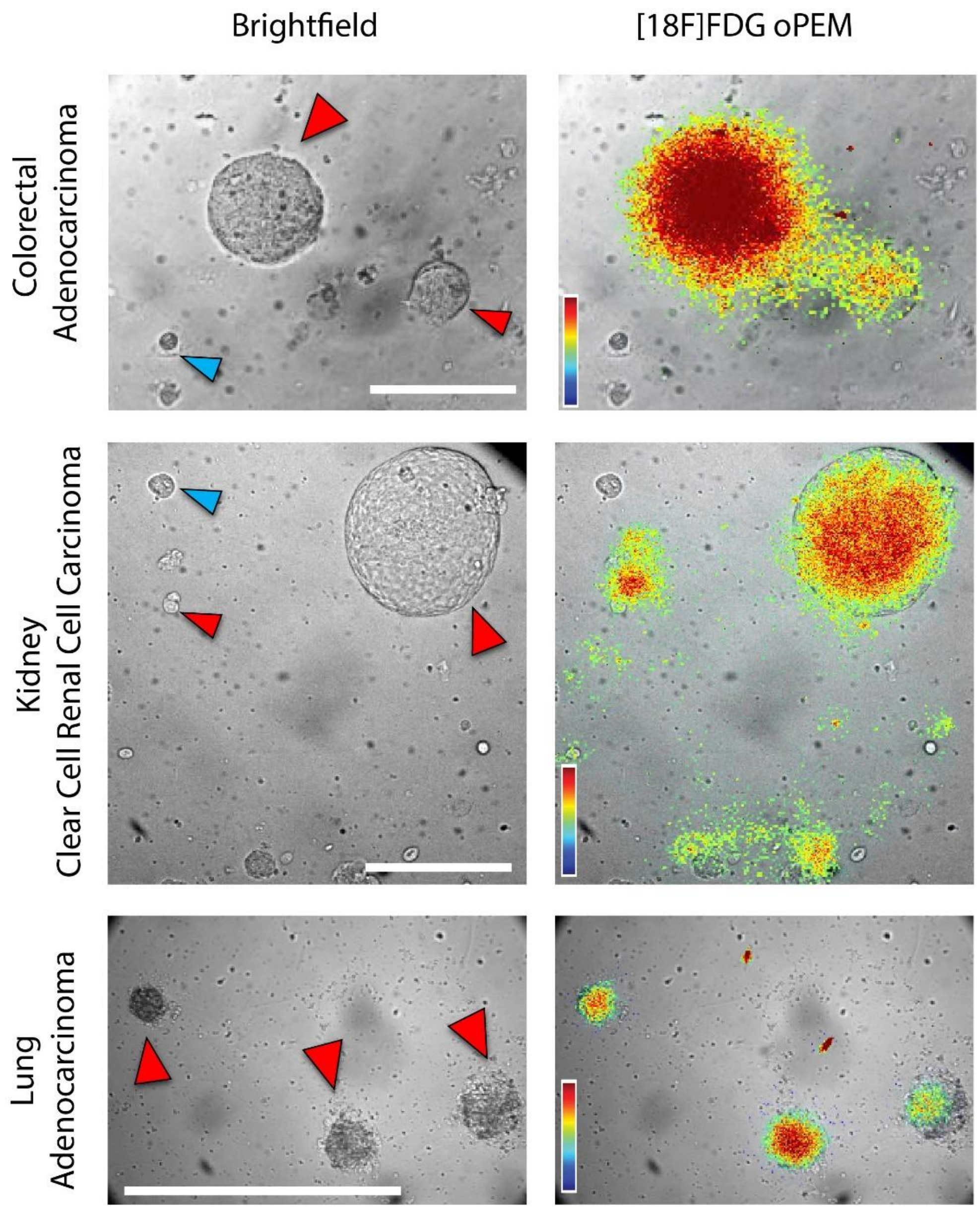

Supplementary Figure 6: In-situ oPEM imaging of organoids inside the Matrigel culture matrix. Organoid models of (a) colorectal adenocarcinoma, (b) clear-cell renal-cell carcinoma, and (c) lung adenocarcinoma demonstrate heterogeneity in organoid size (left panels) and FDG uptake (right panels). The organoid structures were not disturbed and kept inside the Matrigel dome during oPEM imaging. GAGG:Ce scintillators (15 mm diameter, 0.1 mm thickness) were placed over the Matrigel dome, enclosing the culture in a thin layer. Multiple organoids of different sizes are visible in the field of view. FDG-avid and non-avid organoids are shown by red and blue arrowheads, respectively. The measured FDG uptake can be slightly biased in this method if the distances of those organoids to the scintillator

varies significantly. EMCCD exposure time: 120 s, Color bar shows radioactivity (Bq/pixel). Scale bar: 1 mm.

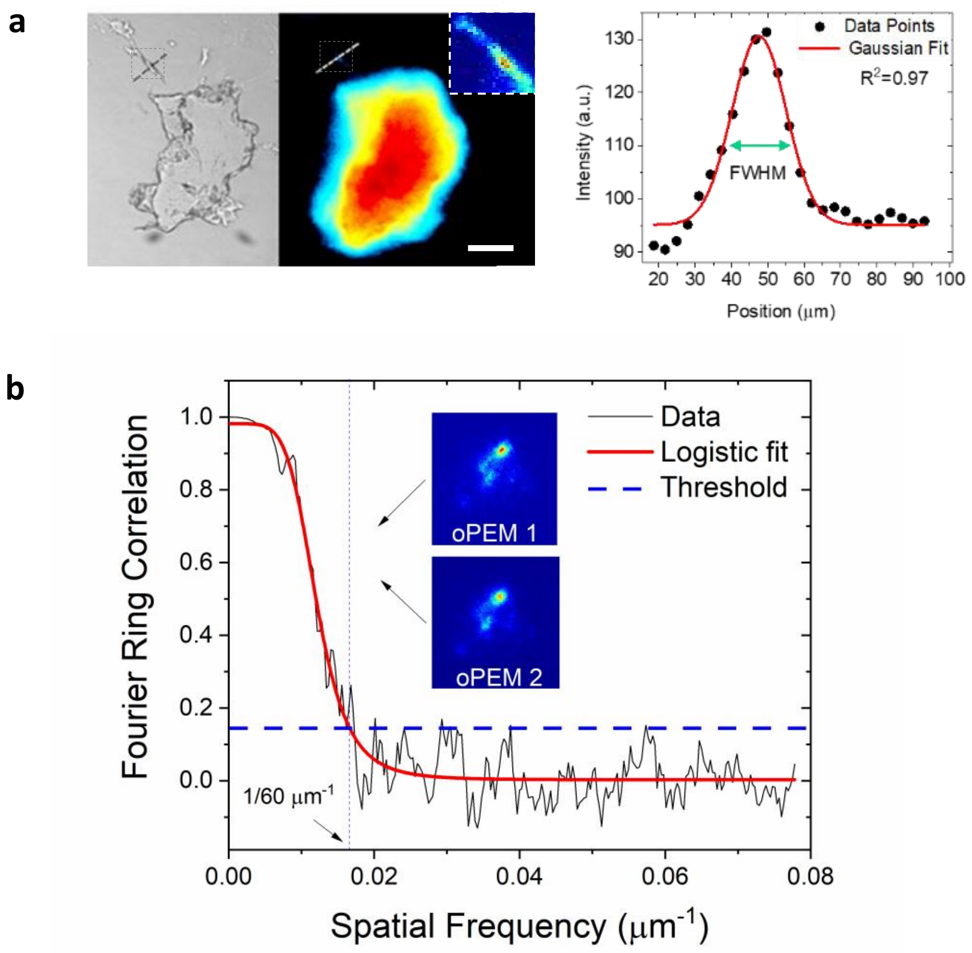

Supplementary Figure 7. Spatial resolution of oPEM. (a) Left: Brightfield and oPEM imaging of an organoid (from patient S1) showing a tiny protrusion-like feature. Scale Bar: 0.1 mm. The inset shows a magnified image of the small structure. Right: Intensity profile through radioluminescence image along the dashed line. FWHM was calculated to be 17.5 ± 0.8 μm along the dotted line. (b) Fourier ring correlation (FRC) analysis of oPEM images. The FRC measures the spatial cross-correlation between two oPEM images of the same organoid sample (inset) over concentric frequency rings. Based on the standard FRC threshold of 1/7 (dashed line, blue), the average spatial resolution of the imaging system is approximately 60 μm. The uncertainty range is ± 4 μm according to the 95 % confidence interval for a logistic fit.

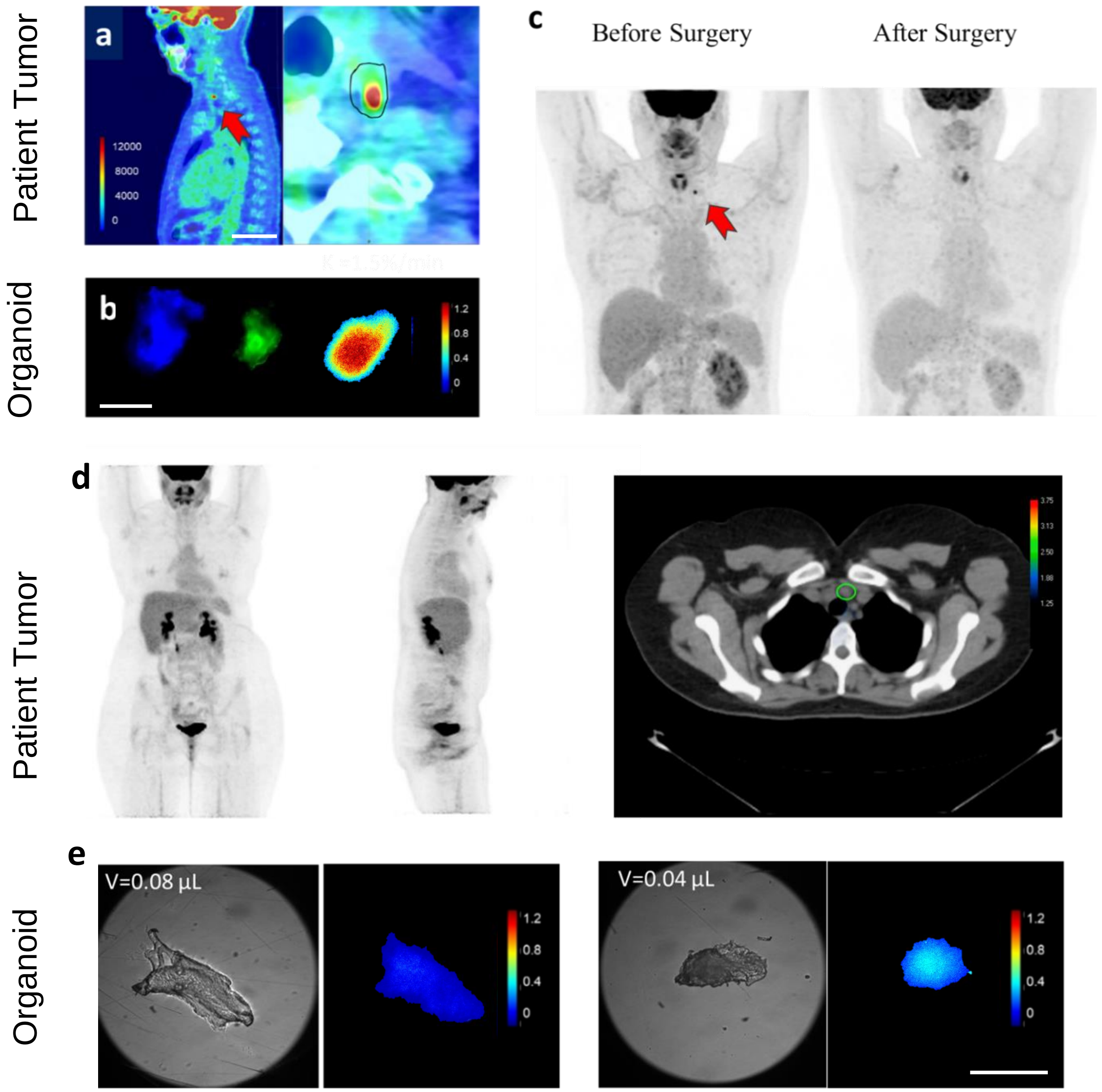

Supplementary Figure 8. Comparison between FDG uptake in organoid and patient-of-origin tumor for patient T1 and T2. (a) PET image of T1. The red arrow points to a cervical lymph node metastatic lesion from thyroid cancer, which is also shown magnified in the right panel. Scale bar: 10 cm. Color bar: Bq/ml (b) oPEM imaging of tumor organoid grown from the same tumor of patient T1. Scale bar: 0.5 mm. Color bar: Bq/pixel. (c) PET scan shows no FDG avid lesion after surgery of T1. (d) PET scan of T2 shows no abnormal uptake as the metastases were not FDG avid. (e) Two oPEM images show organoids derived from the excised lymph nodes metastases with moderate FDG uptake. Scale bar: 1 mm. Color bar shows radioactivity (Bq/pixel)..

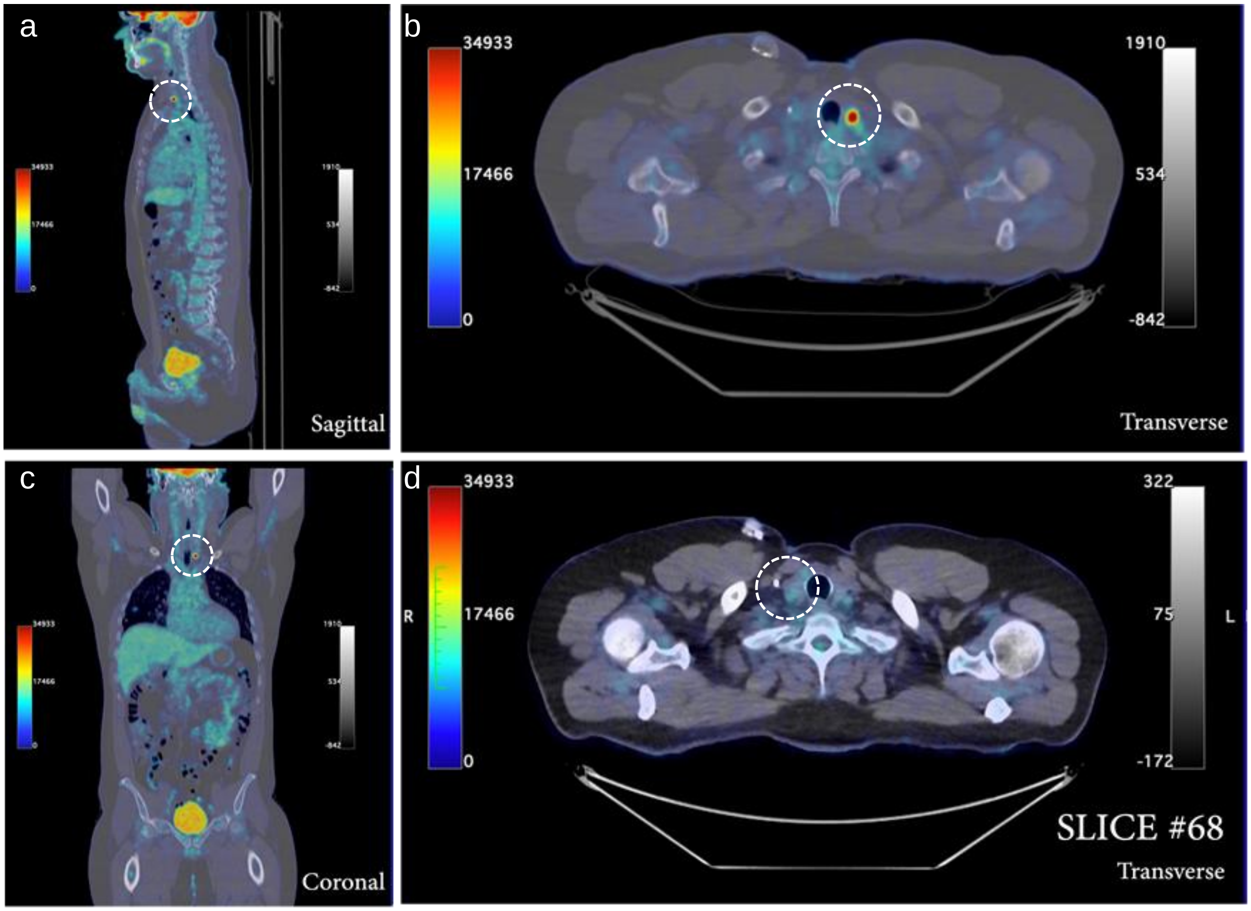

Supplementary Figure 9. PET/CT images of patient T3. (a-c) Sagittal, transverse and coronal PET images show FDG-avid nodule in the left side of the thyroid gland. (d) A different slice from the same PET scan, centered on the location of a second thyroid nodule, on the right side of the thyroid gland, which did not take up any FDG.

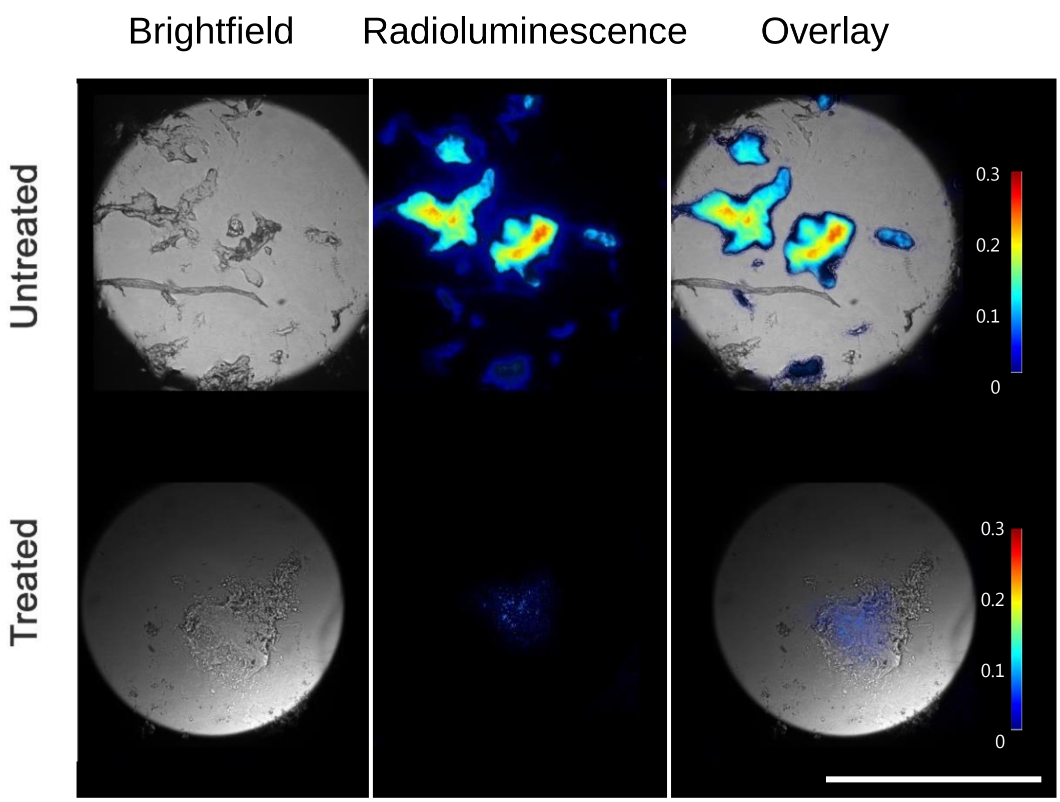

Supplementary Figure 10. oPEM images of organoids 24 h after cisplatin treatment (10 μM dose), compared to untreated organoids. The significant drop of FDG uptake indicates a decline in glucose metabolism and cell proliferation. Scale bar: 1 mm. Color bar shows radioactivity (Bq/pixel).

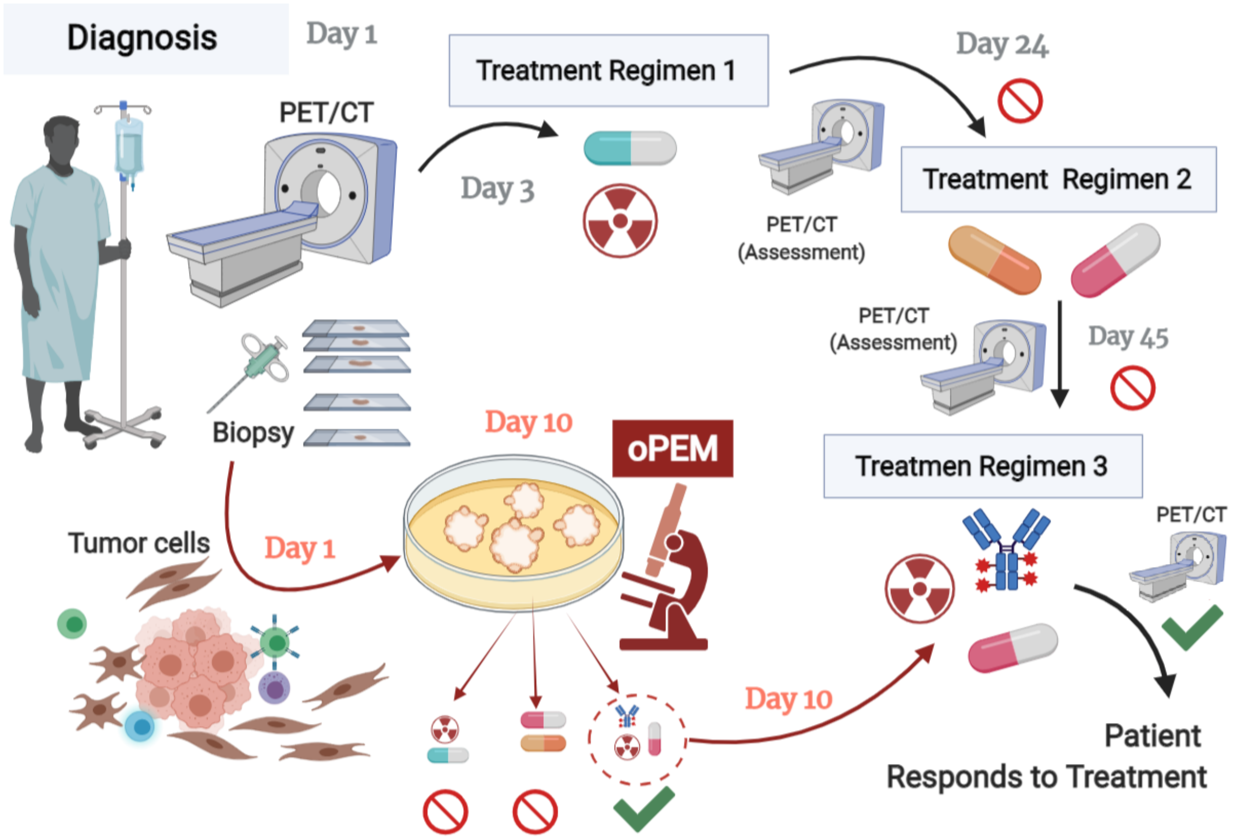

Supplementary Figure 11: Conceptual use of oPEM for individualized second-line therapy. Black arrows (clockwise): Treatment workflow shown for a cancer patient after diagnosis of the disease. In the conventional workflow, the patient fails to respond to treatment regimen 1 and 2 but eventually responds to regimen 3, albeit after a significant delay. Red arrows (counterclockwise): Proposed treatment workflow utilizing oPEM to identify the most effective therapy when the first-line therapy fails. After diagnosis of the disease, tumor organoids are derived in the laboratory from biopsy or surgically resected tissue. Imaging with oPEM is used to predict response to various therapies to select the treatment regimen most likely to elicit a response in the patient. In the future, this workflow can assist physicians in their clinical decisions.

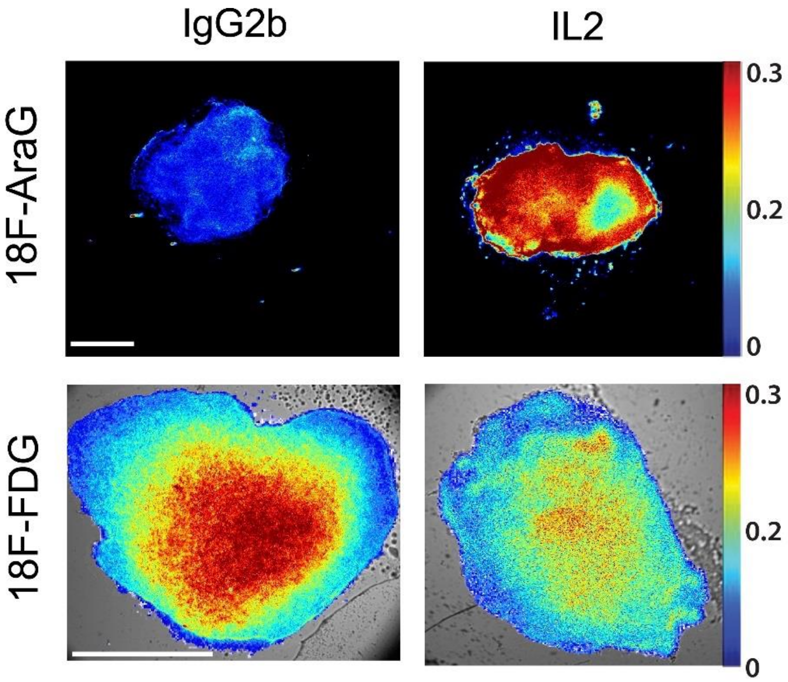

Supplementary Figure 12: oPEM imaging of T-cell activation in patient-derived squamous cell carcinoma organoids. Organoids were cultured using an air-liquid interface (ALI) to preserve tumor immune-microenvironment. To measure immune activation, the organoids were imaged using a novel PET radiotracers, 2'-deoxy-2'-[(18)F]fluoro-9-β-D-arabinofuranosylguanine (^{18}F-AraG. Pilot results show elevated ^{18}F-AraG uptake after IL2 treatment (1000 IU/m) in organoids from some patients. ^{18}F-FDG uptake was also measured in parallel experiments. These data show the feasibility of imaging immune-PET tracer uptake in organoid cultures using oPEM. Color bar shows radioactivity (Bq/pixel). Scale bar: 1 mm

Table S1: Patient and tumor details

Patient ID	Tumor type	Tumor histology (H&E)
**S1**	Squamous cell carcinoma (pT4aN0, p16 negative). Tumor site: mandible, oral cavity	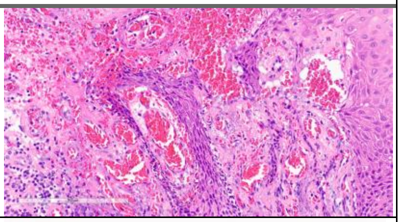
**S2**	Squamous cell carcinoma (pT4aN3b, p16 unknown). Tumor site: tongue, oral cavity	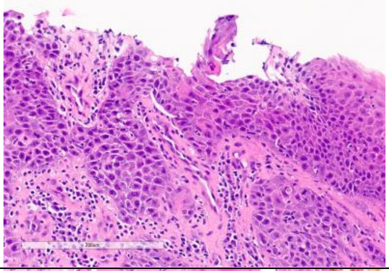
**T1**	Poorly differentiated papillary thyroid carcinoma (pT1a, N1b), metastasis to cervical lymph node	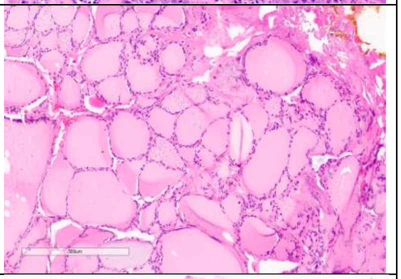
**T2**	Papillary thyroid carcinoma (pT1b, N1b), diffuse sclerosing variant, with extensive lymphovascular invasion	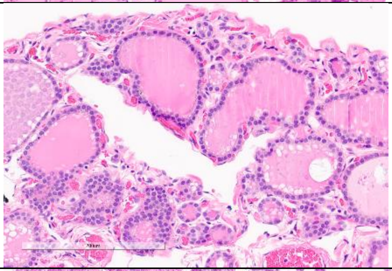
**T3**	Papillary thyroid carcinoma (cT1b, cN0, cM0). Brain metastasis. Sertoli cell tumor with lymphovascular invasion of right testis (rpT1a, pN2, cM0, S0)	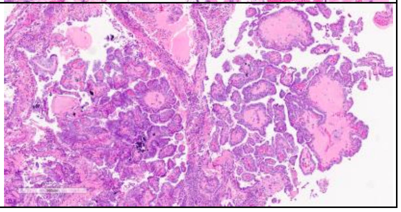

Table S2: Pharmacokinetic parameters for patient and organoid tumors

	Patient ID	FDG Dose (MBq)	Distribu-tion Volume (L)	Mean Plasma Conc. (kBq/mL)	Target Activity (kBq)	Target Volume (mL)	Target Conc. (kBq/mL)	K_i (%/ min)	SUV
**Patient Tumor**	T1	340.4	11.7	16.1	14.4	0.4	36	3.66	8.7
	T2	495.8	12.3	22.5	3.4	0.4	8.4	0.61	1.8
	T3 (R)	407	12.9	17.5	4.8	0.5	9.5	0.89	2.5
	T3 (L)	407	12.9	17.5	61.6	0.5	123.2	11.57	32.4
**Tumor Organoid**	T1	37	0.001	37000	12.25<br>(0.8)[a]<br>(1.0)[a]	0.00018<br>0.00006<br>0.00010	64,809<br>(12,986)<br>(23,400)<br>(51,183)	1.46<br>(0.29)<br>(0.53)<br>(1.15)	1.75<br>0.35<br>0.63<br>1.38
	T2				4.7<br>(2.6)[a]<br>2.9	0.00015<br>0.00007<br>0.00010	29,729<br>(39,500)<br>27,865	0.67<br>(0.88)<br>0.62	0.80<br>1.06<br>0.75
	T3 (R)				0.3<br>0.1<br>0.2<br>3.1<br>0.3	0.00051<br>0.00046<br>0.00035<br>0.00052<br>0.00024	539<br>198<br>672<br>6,012<br>1,397	0.01<br>0.01<br>0.02<br>0.14<br>0.03	0.02<br>0.01<br>0.02<br>0.16<br>0.04
	T3 (L)				2.6<br>5.4<br>2.6<br>22.5<br>9.8	0.00055<br>0.00010<br>0.00008<br>0.00020<br>0.00016	47,980<br>52,292<br>32,263<br>114,742<br>59,906	1.08<br>1.18<br>0.73<br>2.58<br>1.35	1.29<br>1.41<br>0.87<br>3.10<br>1.61

[a] Values in parenthesis are directly estimated from oPEM images in the absence of gamma-counting data
