# Supplementary material for: High-resolution positron emission microscopy of patient-derived tumor organoids
Source: Nat Commun. 2021 Oct 7;12:5883. doi: 10.1038/s41467-021-26081-6 (PMC8497512; doi:10.1038/s41467-021-26081-6)
Supplement: Supplementary file 3 — Reporting Summary [file 41467_2021_26081_MOESM3_ESM.pdf]

nature research

Corresponding author(s): Guillem Pratx

Last updated by author(s): Jul 16, 2021

Reporting Summary

Nature Research wishes to improve the reproducibility of the work that we publish. This form provides structure for consistency and transparency in reporting. For further information on Nature Research policies, see our Editorial Policies and the Editorial Policy Checklist.

Statistics

For all statistical analyses, confirm that the following items are present in the figure legend, table legend, main text, or Methods section.

n/a	Confirmed	
☐	☒	The exact sample size (*n*) for each experimental group/condition, given as a discrete number and unit of measurement
☐	☒	A statement on whether measurements were taken from distinct samples or whether the same sample was measured repeatedly
☐	☒	The statistical test(s) used AND whether they are one- or two-sided<br>*Only common tests should be described solely by name; describe more complex techniques in the Methods section.*
☒	☐	A description of all covariates tested
☒	☐	A description of any assumptions or corrections, such as tests of normality and adjustment for multiple comparisons
☐	☒	A full description of the statistical parameters including central tendency (e.g. means) or other basic estimates (e.g. regression coefficient) AND variation (e.g. standard deviation) or associated estimates of uncertainty (e.g. confidence intervals)
☐	☒	For null hypothesis testing, the test statistic (e.g. F, t, r) with confidence intervals, effect sizes, degrees of freedom and P value noted<br>*Give P values as exact values whenever suitable.*
☒	☐	For Bayesian analysis, information on the choice of priors and Markov chain Monte Carlo settings
☒	☐	For hierarchical and complex designs, identification of the appropriate level for tests and full reporting of outcomes
☐	☒	Estimates of effect sizes (e.g. Cohen's d, Pearson's r), indicating how they were calculated

Our web collection on statistics for biologists contains articles on many of the points above.

Software and code

Policy information about availability of computer code

Data collection	oPEM data were collected using MicroManager 1.4.22 software. DICOM PET/CT images were imported from the Stanford PACS. Some processed PET/CT images were imported directly from the electronic health record (Epic).
Data analysis	ImageJ 1.52a (NIH) and MATLAB R2020b were used to process and analyze the raw images. Some RLM images were reconstructed using ORBIT 1.11. OriginLab 2019b was used for graphical analysis. DICOM images were visualized and processed in Osirix 12.0.3.

For manuscripts utilizing custom algorithms or software that are central to the research but not yet described in published literature, software must be made available to editors and reviewers. We strongly encourage code deposition in a community repository (e.g. GitHub). See the Nature Research guidelines for submitting code & software for further information.

Data

Policy information about availability of data

All manuscripts must include a data availability statement. This statement should provide the following information, where applicable:
- Accession codes, unique identifiers, or web links for publicly available datasets
- A list of figures that have associated raw data
- A description of any restrictions on data availability

The main data supporting the results in this study are available within the paper and its Supplementary Information. The raw microscopy images (oPEM, brightfield and fluorescence) are deposited in a public data repository (doi:10.5061/dryad.63xsj3v2v).

Field-specific reporting

Please select the one below that is the best fit for your research. If you are not sure, read the appropriate sections before making your selection.

☒ Life sciences ☐ Behavioural & social sciences ☐ Ecological, evolutionary & environmental sciences

For a reference copy of the document with all sections, see nature.com/documents/nr-reporting-summary-flat.pdf

Life sciences study design

All studies must disclose on these points even when the disclosure is negative.

Sample size	No specific ample-size calculations were performed, because the goal of this study was to demonstrate a new methodology. We performed oPEM imaging from a total of 6 patients. For comparative analysis of PET and PEM, we choose three patients where the PET data were available. These patients derived sample were sufficient to show a proof-of-principle for oPEM imaging of various canacer organoids using multiple radiotracers used in the clinic.
Data exclusions	No data was excluded from this manuscript.
Replication	The contrast and spatial resolution of oPEM imaging was successfully replicated for >10 SCC organoids and >10 PTC organoids for a total of 6 patients using our established protocol. The Ki calculation and comparison were performed with 3-5 organoids, each for 3 patients or 4 tumor of origin. The response of organoids derived from the cisplatin-resistant patient has not been yet replicated with other patients due to limited availability of cisplatin-resistant patient tissue. We added this data to show a proof-of-concept for screening drug response.
Randomization	There were no experimental and control groups to randomize. We used the exact same methodology for all the samples used in this study. We have tested and compared our results between previously known groups, e.g. between FDG-hot nodules and FDG-cold nodules, or Cisplatin-responsive patient and Cisplatin-resistant patient.
Blinding	Not applicable. The study did not have endpoints for which blinding would be required. Patient-derived organoids were used as a model-system to demonstrate feasibility of oPEM imaging with clinical radiotracers .

Reporting for specific materials, systems and methods

We require information from authors about some types of materials, experimental systems and methods used in many studies. Here, indicate whether each material, system or method listed is relevant to your study. If you are not sure if a list item applies to your research, read the appropriate section before selecting a response.

Materials & experimental systems

n/a	Involved in the study	
☐	☒	Antibodies
☐	☒	Eukaryotic cell lines
☒	☐	Palaeontology and archaeology
☒	☐	Animals and other organisms
☐	☒	Human research participants
☒	☐	Clinical data
☒	☐	Dual use research of concern

Methods

n/a	Involved in the study	
☒	☐	ChIP-seq
☒	☐	Flow cytometry
☒	☐	MRI-based neuroimaging

Antibodies

Antibodies used	Primary antibodies: anti-E-cadherin (mouse anti-human/mouse; BD, # 610181, 1:1000), anti-vimentin (chicken anti-human/mouse; Millipore, # AB1620, 1:1000), and anti-CD3 (rabbit anti-human; Dako, # A0452, 1:100).<br><br>Secondary antibodies: IgG (H+L)-Texas Red (goat anti-mouse; Thermo Fisher, # T-862, 1:1000), IgY (H+L)-AF488 (goat anti-chicken; Thermo Fisher, # A-11039, 1:1000), and IgG (H+L)-Cy5 (goat anti-rabbit; Thermo Fisher, # A10523, 1:1000).
Validation	The antibodies used for immuno-fluorescence imaging were stated to be validated by the respective manufacturers. Negative control (without primary antibody) was used to confirm the specificity of IF staining prior to experiment. The IF staining protocol was reproduced across specimens within the same species.<br><br>Further details on validation can be found in the manufacture's websites:<br>https://www.thermofisher.com/us/en/home/life-science/antibodies/invitrogen-antibody-validation.html<br>https://www.sigmaaldrich.com/US/en/technical-documents/technical-article/protein-biology/elisa/antibody-standard-validation<br>https://www.biocompare.com/Reproducibility/336622-Antibody-Validation/

Eukaryotic cell lines

Policy information about cell lines

Cell line source(s)	Control MDA-MB-231 cells were purchased from Cell BioLabs (Feb 2016). NIS-transfected NDA-MB-231 cells were provided by the lab of Dr. Irene Wapnir.
Authentication	Control Authenticated by the vendor in 2016. The NIS-transfected NDA-MB-231 cells were not authenticated but displayed expected phenotype of iodine uptake.
Mycoplasma contamination	The cell lines were not tested for mycoplasma contamination
Commonly misidentified lines (See ICLAC register)	No commonly misidentified cell lines were used.

Human research participants

Policy information about studies involving human research participants

Population characteristics	Organoids from 6 adult patients were used in this study (3 head-and-neck squamous cell carcinoma, 3 papillary thyroid cancer). The patients were selected by the surgeons on the basis of sufficient tumor tissue available to grow organoids, clinical characteristics, and availability of PET/CT prior to surgery. The patients include both sexes, had age between 29-70 years and weighted between 59-107 kg.
Recruitment	Patient were invited to participate during their surgical consultation. Any potential self-selection bias is unlikely to impact the results of this study since this was a pilot study aiming to evaluate a new technology. There were no explicit comparisons between patients.
Ethics oversight	Stanford University institutional review board gave approval for this study (protocol #6037). Patients who participated in this study gave informed consent for the use of tissue specimens and clinical data for this research study, and for the publication in scientific articles of the resulting de-identified data. All medical images (PET/CT) published in the manuscript were de-identified to protect the privacy of the patients.

Note that full information on the approval of the study protocol must also be provided in the manuscript.
